# The Housing Question

**Published:** 1903-02-28

**Authors:** 


### The Housing Question.

The question of the better housing of the working classes, intimately connected as it is with everything which makes for the healthiness, the sobriety, the cleanliness, and the self-respect of the persons more immediately concerned, is well worthy of the attention which was directed to it by the debate in the House of Commons on Dr. Macnamara's amendment to the Address; and it was eminently satisfactory to find that this attention was by no means confined to the opponents of the Government, or displayed merely as a weapon to be used against political adversaries, and to be cast away when it had served its turn. Even on the Ministerial side of the House candid friends were not wanting; and it was rendered apparent that no Ministry of the near future, however constituted or whatever doctrines it may profess, will be able to escape from the necessity of at least appearing to regard a better supply of labouring-class dwellings as among the objects to the attainment of which its legislative or administrative activity should be directed. There is, it cannot be denied, an altogether admirable amount of zeal and enthusiasm for reform in the abstract; but the President of the Local Government Board had an easy task in showing that this zeal is very apt to evaporate whenever any endeavour is made to secure its practical application to any individual case. The Board is roundly abused for its alleged remissness in the enforcement of the law; but, as soon as a local authority becomes the recipient of official remonstrance, the member of Parliament for the district concerned is apt to find his way to Whitehall, and not only to urge that the district council, or whatever it may be, of his constituency should be left to conduct their business in their own way, and to leave it unconducted if it so please them, but to express preparedness, if the offensive remonstrance be not withdrawn, to bring the matter before the House on a motion for the reduction of the President's salary. Not only, therefore, is there some want of sincerity in the common outcry about housing, but there is also a very manifest tendency towards that exaggeration of existing evils with which insincerity is apt to be accompanied.

The gratifying circumstance to which we called attention last week, that the death rate for England and Wales in 1902 had been only 16·3 per 1,000, may be taken, we think, to prove that the sanitary evils connected with bad housing, and therefore, presumably, the badness of the housing itself, are in course of steady diminution, and that hence at least some portion of the present outcry may be attributed to an increasing sense of the magnitude of the evil, rather than to any actual increase in its proportions. If this be so, it is likely to be ameliorated, and eventually cured, by the gradual spread of enlightenment and intelligence, and the school, if the new Education Act be only properly worked, may become a potent agent for good as far as the rising generation is concerned. At present, we apprehend, large numbers of the working classes are quite content to live in pigsties, and feel comparatively little interest in the question. The honourable member for Bethnal Green told the House of Commons that in the East of London the "pauper aliens" soon converted the newest and most approved dwellings into insanitary tenements, and that the model houses put up by the County Council on the Boundary Street area were now full of aliens, who lived in the most undesirable conditions. Whether true or not of the aliens, something of the same kind was undoubtedly true, a few years ago, of born Londoners, who too often defeated the good intentions of landlords by the wanton destruction of arrangements conducive to cleanliness and decency. Could not the County Council itself do a great deal towards reform if it would promptly expel from its houses all tenants who were manifestly unfit to occupy them, and so let it be understood that a Council tenant might, indeed, be a poor man, but that he must, to the extent of his opportunities, be a clean and decent one, taking reasonable care of the property which he was permitted to occupy? In point of fact, the question is very largely one of cleanliness; and the school and the County Council together might effect a revolution in the habits of the working classes of London in this respect, and might bring them up to some approach to the American standard. The County Council admits into its tramcars, without hindrance, men in filthy working clothes whose faces and hands are black with grease and grime, and who both leave stains upon any seat which they occupy and soil in a corresponding manner the clothes of any passenger who either succeeds them or is pressed against them. No American workman would show himself in public in such a condition; and if the Council and the school would practically teach adults and children that personal filth is disgraceful, an increased demand for decent houses would before long follow from a demand for personal cleanliness.

